# 3D-printing-assisted fabrication of hierarchically structured biomimetic surfaces with dual-wettability for water harvesting

**DOI:** 10.1038/s41598-023-37461-x

**Published:** 2023-07-01

**Authors:** Yeongu Choi, Keuntae Baek, Hongyun So

**Affiliations:** 1 Department of Mechanical Engineering, Hanyang University, Seoul, 04763 South Korea; 2 Institute of Nano Science and Technology, Hanyang University, Seoul, 04763 South Korea

**Keywords:** Mechanical engineering, Surface patterning

## Abstract

Freshwater acquisition methods under various environments are required because water scarcity has intensified worldwide. Furthermore, as water is an essential resource for humans, a freshwater acquisition method that can be utilized even under harsh conditions, such as waterless and polluted water environments, is highly required. In this study, a three-dimensional (3D) printing-assisted hierarchically structured surface with dual-wettability (i.e., surface with both hydrophobic and hydrophilic region) for fog harvesting was developed by mimicking the biological features (i.e., cactus spines and elytra of Namib Desert beetles) that have effective characteristics for fog harvesting. The cactus-shaped surface exhibited self-transportation ability of water droplet, derived from the Laplace pressure gradient. Additionally, microgrooved patterns of the cactus spines were implemented using the staircase effect of 3D printing. Moreover, a partial metal deposition method using wax-based masking was introduced to realize the dual wettability of the elytra of the Namib Desert beetle. Consequently, the proposed surface exhibited the best performance (average weight of 7.85 g for 10 min) for fog harvesting, which was enhanced by the synergetic effect between the Laplace pressure gradient and surface energy gradient. These results support a novel freshwater production system that can be utilized even in harsh conditions, such as waterless and polluted water environments.

## Introduction

Currently, water scarcity has intensified because the existing water supply has not satisfied the demand for water owing to the increase in global population^1^. Furthermore, although water is an essential resource for human life, the amount of freshwater available to humans depends on the geographic location (e.g., river, desert, and ocean) and the water supply infrastructure^2,3^. To address this problem, several works to obtain freshwater, such as fog harvesting^4–6^, desalination^7,8^, membrane filtration^9,10^, and solar-driven water production^11^, have been conducted. Fog harvesting, which can capture atmospheric water, is an attractive method that can be used even in arid (e.g., deserts) and polluted water environments^12^. Moreover, compared with other methods, fog harvesting has the advantage of not only having no location restrictions but also rapid on-site production^13^. In nature, various biological features to obtain freshwater in harsh environments can be found, such as cactus spines^14^, silk of spider^15^, elytra of Namib Desert beetle^16^, and inner wall of nepenthes^17^. Cacti and Namib Desert beetles are representative desert creatures that collect water from the atmosphere in harsh environments. Cactus can effectively collect water from fog in the desert using microgrooved patterns on its spines^18^. Specifically, the spines of the cactus have self-water transportation and water collection abilities derived from the Laplace pressure and wettability gradients^19^. The Laplace pressure gradient allows the collected droplets to be self-transported from the tip to the base of the spine^20^. Additionally, the wettability gradient derived from the grooved surface enhances the surface wettability (e.g., hydrophilicity and hydrophobicity)^14^. Moreover, the Namib Desert beetle has water collection and self-transportation abilities induced by the surface energy gradient from the dual wettability (wax-coated hydrophobic valleys and hydrophilic bumps) on the elytra surface^21^. Water droplets from fog preferentially condense on hydrophilic bumps, whereas water droplets condensed on hydrophobic valleys are transported to hydrophilic bumps via a surface energy gradient^22^.

Inspired by these biological features, various surfaces for fog harvesting have been designed to mimic the elytra of the Namib Desert beetle^23–26^ or the spine of the cactus^27–29^. To manufacture a fog-harvesting surface inspired by the elytra of the Namib Desert beetle, methods to realize coexisting hydrophobic and hydrophilic regions have been demonstrated using taping^30,31^, film masking^32^, and inkjet printing^33^. Because taping and film masking require additional masks, these methods are not suitable for mass production if the masking area has a complex morphology. Although inkjet printing does not require a mask, it cannot be applied to complex morphologies. Manufacturing methods to fabricate fog-harvesting surfaces inspired by the spines of cacti have been recently demonstrated using laser drilling^34^, CNC machines^35^, immersed surface accumulation-based three-dimensional (3D) printing^36^, and photolithography^37^. Although the morphology of the cactus spine is well understood, most methods involve high costs, complicated processes, and specialized equipment. Moreover, most fog-harvesting surfaces inspired by the cactus spine mimic only the conical structure, except for the microgrooved patterns of the cactus spine. Unlike the aforementioned manufacturing methods, fused filament fabrication (FFF)-type 3D printing technology offers the advantages of low cost, rapid prototyping, and simple manufacturing^38^. However, FFF-type 3D printing has a chronic drawback of the staircase effect caused by stacked filaments^39^. Therefore, hierarchically structured surfaces with various designs can be effectively realized using the staircase effect of FFF-type 3D printing.

**Figure 1:**
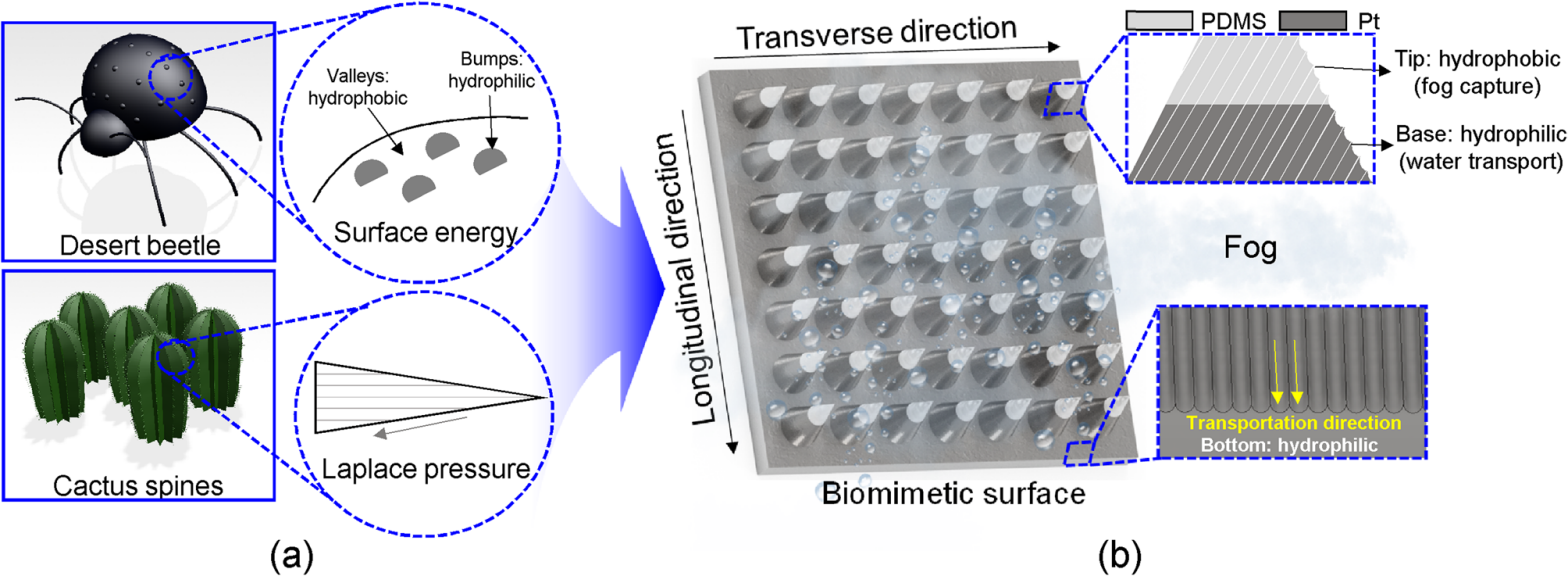
(**a**) Illustration of the characteristics of both elytra of the Namib Desert beetle and cactus spines; (**b**) schematic of the hierarchically structured biomimetic surfaces inspired by the elytra of the Namib Desert beetle and cactus spines.

In this study, a novel manufacturing method for hierarchically structured surfaces with dual wettability was demonstrated for fog (water) harvesting applications. The hierarchically structured surface for water harvesting was designed by mimicking not only the conical structure with a microgrooved pattern of the cactus spine, but also the dual-wettability of the elytra of the Namib Desert beetle, as illustrated in Fig. 1. A macro–micro hierarchically structured surface was cast in a polylactic acid (PLA) mold printed using an FFF-type 3D printer. A microgrooved pattern of the cactus spine was implemented on the proposed surface using the staircase effect of FFF-type 3D printing. Platinum was directly deposited on the oxygen plasma-treated polymer surface for hydrophilic modification. To prevent the hydrophilic modification of the tip region of hierarchically structured surfaces, a paraffin wax-based masking method that can be applied to mass production was introduced. To demonstrate the fog-harvesting performance of the proposed surfaces and the effect of the microgrooved pattern on fog harvesting, six different types of surfaces were prepared and compared with two different micropatterned directions under identical conditions. Furthermore, three different conical structure diameters of the proposed surfaces were compared to demonstrate the effect of the combination of Laplace pressure and surface energy gradient.

### Design and fabrication

**Figure 2:**
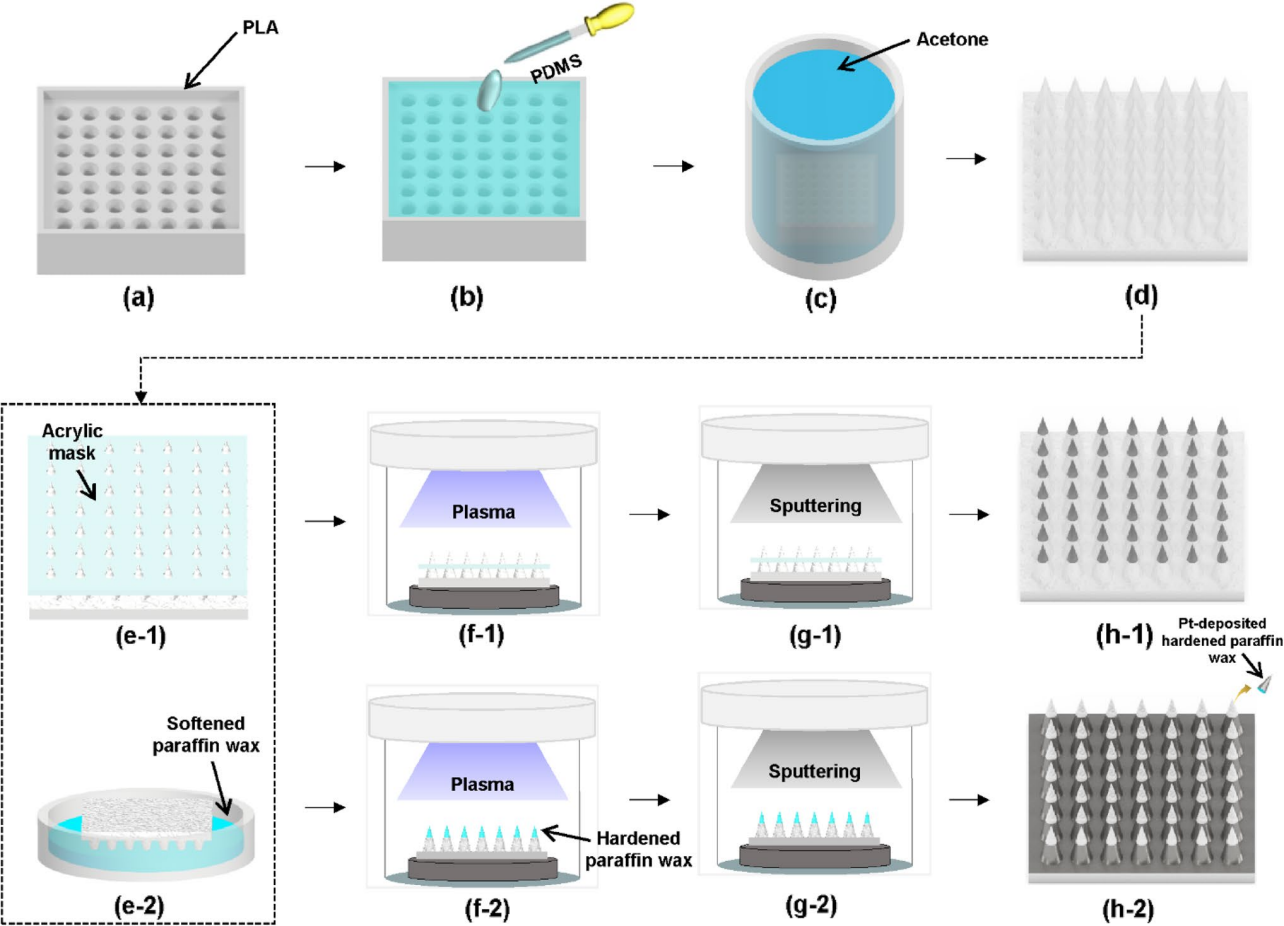
Schematic of the overall fabrication process. (**a**) Printing PLA mold with an 80° tilted angle; (**b**) Pouring PDMS onto the PLA mold and curing; (**c**) Immersing PLA mold in acetone; (**d**) Demolding the hierarchically structured PDMS surface; (**e-1**) Putting the acryl mask on the hierarchically structured surface; (**e-2**) Dipping the tip part in the softened paraffin wax; (**f-1**), (**f-2**) Oxygen plasma treatment; (**g-1**), (**g-2**) Pt sputtering; (**h-1**) Hierarchically-structured surface with Pt deposited on the tip part; (**h-2**) Hierarchically-structured surface with Pt deposited without the tip part.

To demonstrate the effects of wettability and hierarchical structures, six different types of water-harvesting surfaces were designed and defined (Table S1 in Supplementary Information). The overall fabrication method for the hierarchically structured surfaces (i.e., CO, TI, and BI) is illustrated in Fig. 2. To cast the conical array polymer surface, a predesigned mold (dimensions: 32 mm × 32 mm × 8 mm) was first printed with a PLA filament (Ø1.75 mm) using an FFF-type 3D printer (3DWOX 2X, Sindoh), as shown in Fig. 2a. The 3D printing conditions, such as nozzle diameter, printing speed, extruder temperature, stage temperature, printing angle, and layer height, were set to 0.4 mm, 40 mm/s, 200 ℃, 60 ℃, 80°, and 0.1 mm, respectively. Subsequently, the polydimethylsiloxane (PDMS) mixture (prepolymer: curing agent = 10:1) was poured onto the prepared mold, as shown in Fig. 2b. The PDMS mixture in the mold was degassed for 30 min and cured in an oven at 60 $\mathrm{^\circ{\rm C} }$ for 6 h. Then, the fully cured PDMS surface was detached from the PLA mold by immersing in acetone for 4 h, as shown in Fig. 2c. The PDMS surfaces with hierarchically structured patterns can be demolded without damage by immersing in acetone. Figure 2d shows a hierarchically structured PDMS surface (i.e., CO) inspired by the cactus spine, which is a well-known representative water collection structure. An acrylic mask containing 3 mm-diameter circular holes at a distance of 1 mm was patterned using a laser cutter (IS-1290, INNOSTA). An acrylic mask was inserted into the conical structure to prevent Pt deposition on the base and bottom parts, as shown in Fig. 2e-1. To prevent Pt deposition on the tip, paraffin wax was introduced as a mask to cover the conical structure of CO. The melted paraffin wax was prepared by heating the solid wax at 80 $\mathrm{^\circ{\rm C} }$ for 40 min. The CO tip was then dipped five times for each 5 s in softened paraffin wax, as shown in Fig. 2e-2. Subsequently, the paraffin wax mask covering the conical structure of the CO was fully solidified for 30 min at room temperature. To clean the PDMS surface and enhance the adhesion between the Pt and PDMS surfaces, the hierarchically structured PDMS surfaces with the acrylic mask and hardened paraffin wax on the tip were subjected to oxygen plasma treatment at 100 W for 1 min (Fig. 2f-1 and 2f-2). Next, a 60 nm-thick Pt layer was deposited on the masked PDMS surfaces using a high vacuum sputter coater (CCU-010 HV, Safematic), as shown in Fig. 2g-1 and 2g-2. Then, the acrylic mask and paraffin wax were removed to realize a hierarchically structured PDMS surface with only Pt deposited on the tip (i.e., TI) and the base and bottom parts (i.e., BI), respectively. Finally, hierarchically structured PDMS surfaces with dual hydrophobic–hydrophilic wettability inspired by the cactus spine and Namib Desert beetle were demonstrated, as shown in Fig. 2h-1 and 2h-2. Additionally, a hydrophilic PDMS surface with a hierarchical structure (i.e., CI) was prepared by Pt deposition after oxygen plasma treatment without a mask. Furthermore, a flat hydrophobic PDMS surface (i.e., FO) was prepared by casting it in an unpatterned mold (32 mm × 32 mm × 3 mm). To prepare a flat hydrophilic PDMS surface (i.e., FI), the FO was treated with oxygen plasma followed by Pt deposition.

## Results and discussion

To demonstrate the water collection performances of the prepared different types of surfaces, an experimental setup that can prevent the influence of the external environment was established, as depicted in Fig. S1 in Supplementary Information. In particular, the prepared testbed considered the angular position of the surface to minimize the gravitational effect of the droplets during the fog collection^40–42^. The detailed information of experimental setup and surface analysis method is shown in I. Experimental setup section, II. Surface analysis section, and Fig. S2 in Supplementary Information. In addition, the details of prepared different types of surfaces are shown in III. Surface and structural characteristics section, Fig. S3, and Table S2 in Supplementary Information.

To characterize the water capture and drainage behavior according to the wettability of the flat surface, optical images of FO and FI were captured using a high-resolution camera until the captured water was drained. As shown in Fig. S4a in Supplementary Information, FO collected more moisture than FI, whereas FI exhibited low moisture collection, coalescence, and drainage times than FO. As water exhibits high surface tension on a hydrophobic surface and low surface tension on a hydrophilic surface, the amount of atmospheric moisture that can reach the surface varies depending on wettability. Therefore, a hydrophobic surface captures more water than a hydrophilic surface in the same area. However, owing to the rapid water saturation properties of hydrophilic surfaces, they are advantageous in terms of water collection speed, coalescence, and drainage compared to hydrophobic surfaces. Hence, FI showed 53 s for the first drainage of the captured water, whereas FO showed 65 s. From the difference in wettability, a surface energy gradient was generated, which can be defined as follows^43^:

1 $$F=\underset{{L}_{tip}}{\overset{{L}_{base}}{\int }}{\sigma }_{w}(\mathit{cos}{\theta }_{a}-\mathit{cos}{\theta }_{r})dL,$$

where ${\theta }_{a}$ is the advancing contact angle of droplets on the middle point of the cone, ${\theta }_{r}$ is the receding contact angle of droplets on the middle point of the cone, ${\sigma }_{w}$ is the surface tension of water, and $dL$ is the integral variable along the length from the tip part to the base part. A detailed schematic of the surface-energy gradient is shown in Fig. S4b in Supplementary Information. In the cone-shaped morphology, a Laplace pressure difference is generated from the tip to the base as follows^14,44^:

2 $$\Delta {P}_{curvature}=-\underset{{r}_{1}}{\overset{{r}_{2}}{\int }}\frac{2{\sigma }_{w}}{{\left(R+{R}_{0}\right)}^{2}}\mathrm{sin}\frac{\alpha }{2}dz,$$

where ${R}_{0}$ is the drop radii, $R$ is the local radius of the cone (${r}_{1}$ and ${r}_{2}$ are the local radius of the cone at the two opposite sides of a drop), $\alpha$ is the angle of the tip, and $dz$ is the incremental radii of the cone. Owing to the pressure difference ($\Delta {P}_{curvature}$) inside the droplet, a driving force is generated that causes the droplet to move along the cactus spine from the tip to the base, as depicted in Fig. S4c in Supplementary Information.

**Figure 3:**
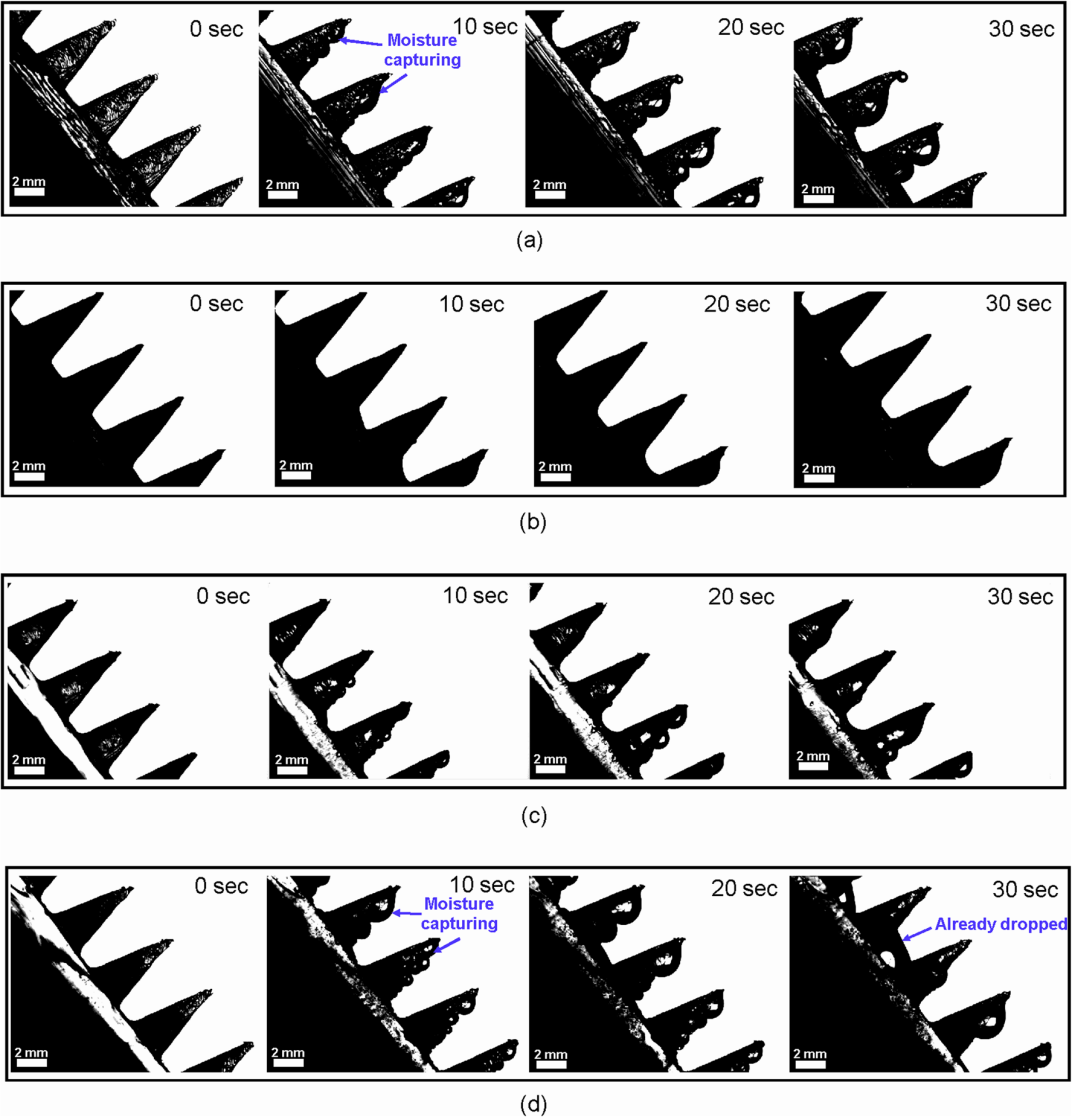
Sequential images of fog capture and water droplet transportation process on the tip and base parts. (**a**) CO; (**b**) CI; (**c**) TI; (**d**) BI.

To demonstrate the water capture and drainage behavior under the effect of different wettability regions on the hierarchically structured surfaces (CO, CI, TI, and BI), side-view optical images of the hierarchically structured surfaces were obtained every 10 s during fog harvesting. As shown in Fig. 3a, CO captured the moisture from the tip. Then, water droplets were transferred to the base but did not reach the bottom region even at 30 s. However, in the case of CI, the water droplets rapidly spread over the entire surface owing to the high surface energy of hydrophilicity. Thus, CI cannot be used to determine the transfer speed from the tip to the base through qualitative analysis, as shown in Fig. 3b. Notably, the hydrophobic surface can capture a large amount of water but has a low transfer rate, whereas the hydrophilic surface can capture a small amount of water but has a high transfer rate of the collected water. Similar to CO, the water droplets captured on the conical structures of the TI did not easily reach the bottom region because of the opposite surface energy gradients, as depicted in Fig. 3c. Additionally, water droplets were mainly captured in the base region rather than the tip region of the TI because of the hydrophilicity of the conical structures of TI. However, BI represents not only a large amount of captured water on the conical structures, but also a high transfer rate of the water droplet from the tip to the bottom region, as shown in Fig. 3d. The hierarchically structured surfaces exhibited the ability to transport water from the tip to the base because of the Laplace pressure gradient derived from the conical structure, as shown in Fig. 3. However, the speed of water transfer from the tip to the base varies in different wettability regions owing to the changes in the surface energy gradient. Thus, the surface gradient can effectively enhance the driving force derived from the Laplace pressure gradient. Therefore, the efficiency of fog harvesting can be enhanced by a hierarchically structured surface with the dual-wettability region of BI (hydrophobicity of the tip region and hydrophilicity of the base and bottom regions).

To investigate the effects of the parallel-aligned microstructured layer of the bottom region during water harvesting, sequential optical images of the hierarchically structured surfaces along two directions (CO-T, CO-L, CI-T, CI-L, TI-T, TI-L, BI-T, and BI-L) were obtained every 12 s. Two directions (-L and -T) were set according to the L and T directions of the aligned microstructured layer toward the water container. As depicted in Fig. S5 in Supplementary Information, the droplets collected in the bottom region of the hierarchically structured surfaces (i.e., CO, CI, TI, and BI) combine and spread along the L direction. In terms of the seven types of the hierarchically structured surfaces (CO-T, CO-L, CI-T, CI-L, TI-T, TI-L, and BI-T), the collected water on the bottom regions was combined at 36 s and spread along the L direction, as shown in Fig. S5a–d in Supplementary Information. However, the collected water in the bottom region of BI-L was combined at 24 s and drained into the water container along the L direction at 36 s, as shown in Fig. S5d in Supplementary Information. It should be noted that the aligned microstructural layers confine the water transportation direction along the L direction, and the gravitational force enhances the coalescence and drainage of droplets along the L-direction. Therefore, hierarchically structured surfaces along the L direction can easily drain water into the water container compared with those along the -T direction.

**Figure 4:**
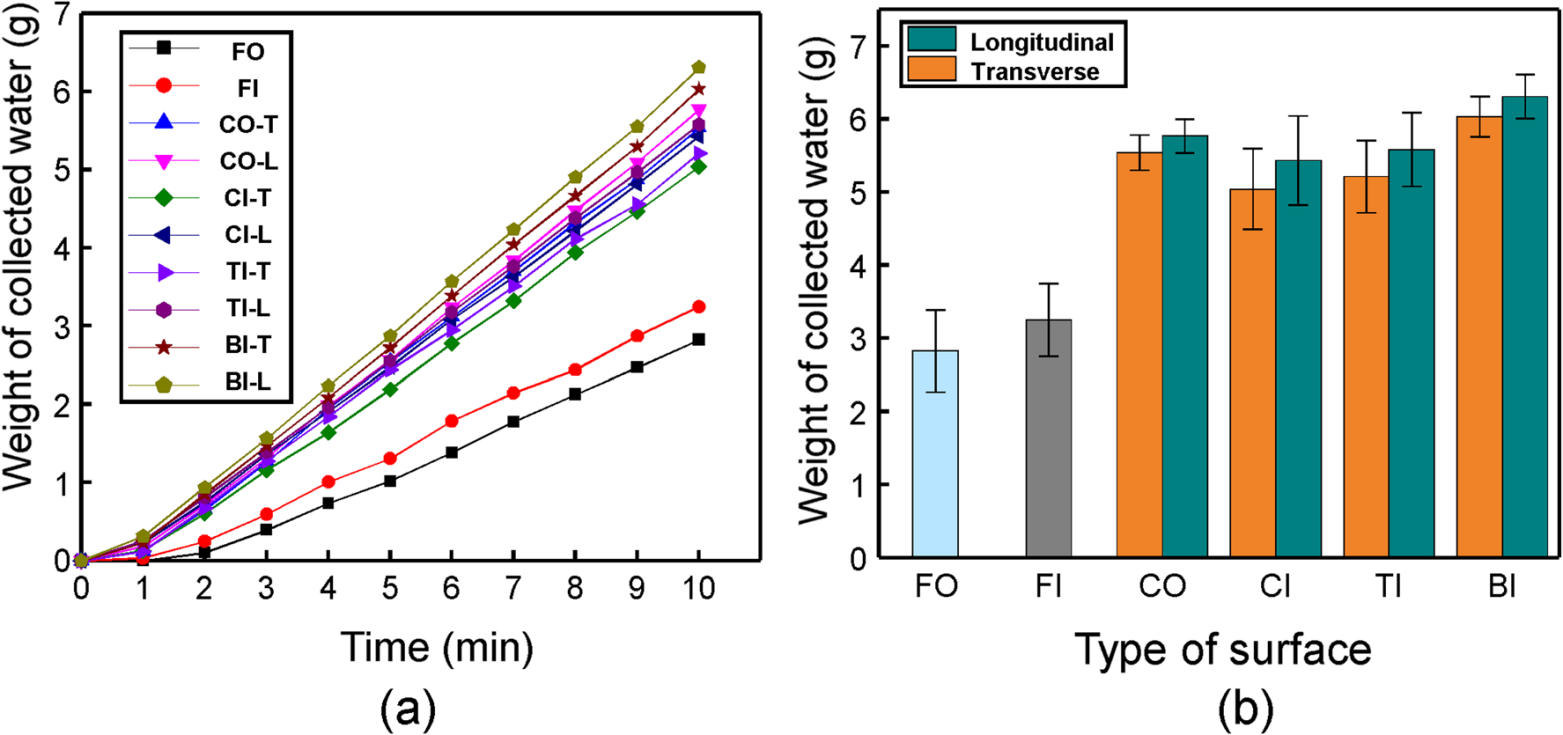
Comparison of water-collecting performance for different types of surfaces along the two directions (-T and -L). (**a**) Time-dependent amount of collected water; (**b**) total amount of collected water for 10 min.

For the quantitative analysis of the proposed surfaces along different directions, the time-dependent weight and total weight of the collected water were obtained, as shown in Fig. 4a,b. The weight of the collected water was measured every 1 min for 10 min on six different surfaces along different directions (FO, FI, CO-T, CO-L, CI-T, CI-L, TI-T, TI-L, BI-T, and BI-L). For flat surfaces, the average weights of the collected water in FO and FI were measured at 2.82 g and 3.25 g, respectively. Although FO can capture more moisture than FI, the fog-harvesting performance of FI was approximately 15% better than that of FO. These results demonstrate that rapid drainage into a water container is an important factor for enhancing the performance of water harvesting. Thus, the hydrophilicity in the bottom region of the hierarchically structured surfaces can be effective for fog harvesting. The fog-harvesting performance of the hierarchically structured surfaces was improved by an increase in the surface area compared to the flat surfaces (FO and FI). Furthermore, the hierarchically structured surfaces along the-L direction (CO-L, CI-L, TI-L, and BI-L) exhibited higher performance in fog harvesting than those along the-T direction (CO-T, CI-T, TI-T, and BI-T) owing to the microstructured layer-assisted water transportation and gravitational force. Consequently, the fog-harvesting performance of BI-L increased by 123% compared to that of FO, as shown in Fig. 4b. BI-L exhibited the highest performance (total collected weight of 6.31 g for 10 min) using the fog-harvesting performance enhancement method (i.e., surface energy gradient, Laplace pressure gradient, hierarchical structure, micro-structured layer assisted water transportation, and gravitational force).

**Figure 5:**
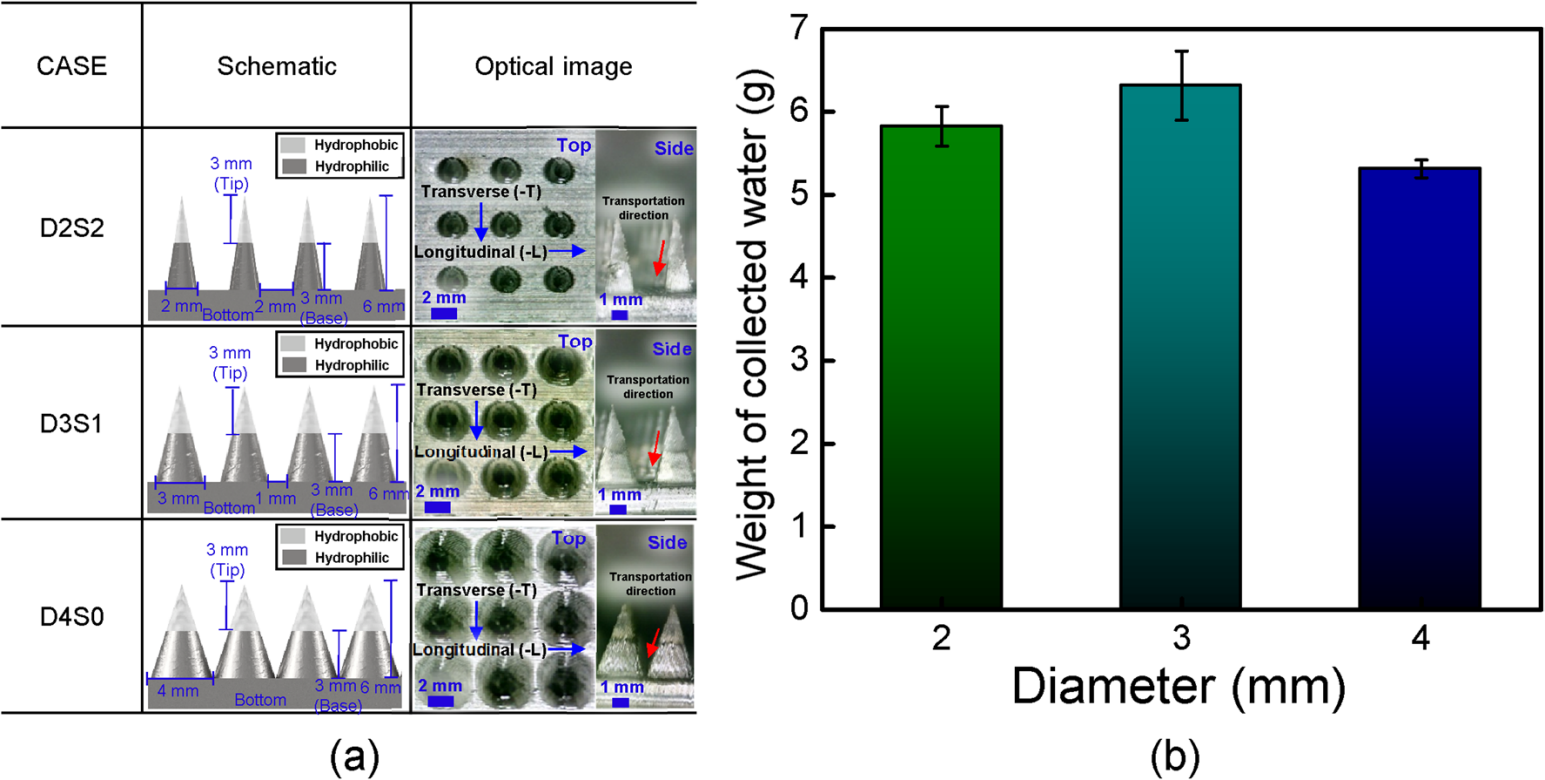
(**a**) Schematic and optical image (top view and side view) of BI-L with fixed number of conical structures; (**b**) average amount of collected water for 10 min of using BI-L with fixed number of conical structures.

To demonstrate the main factors, i.e., Laplace pressure gradient, surface area, and drainage efficiency, of the water harvesting performance of BI-L, three parameters (i.e., different diameter and number of conical structures, and distances between conical structures) of BI-L were considered. To compare and analyze the fog harvesting performance of surfaces by three factors, seven types of surfaces (i.e., D2S1, D2S2, D3S0, D3S1, D3S2, D4S0, and D4S1) were designed and prepared. To ensure identical wettability conditions of the prepared surfaces, the hydrophobic region was fixed at a height of 3 mm in the tip region of the conical structures. The detailed type and geometry information of prepared surfaces to demonstrate the main parameters were listed in Table S3 in Supplementary Information. To compare the performance of surfaces with fixed number of conical structures, three types of BI-L with different diameter of conical structure and distances between conical structures (i.e., D2S2, D3S1, and D4S0) were utilized, as shown in Fig. 5a. Figure 5b depicts the average weight of the collected water of BI-L with conical structures of different diameters. The average weights of collected water of D2S2 and D4S0 were 5.73 g and 5.31 g, respectively. At a fixed height of the conical structures, the Laplace pressure increased as the diameter decreased, whereas the surface area increased as the diameter increased. Hence, although D4S0 has large surface area than other surfaces, D4S0 exhibited the lowest water-harvesting performance owing to its lowest Laplace pressure, whereas D2S2 exhibited a slightly higher performance than D4S0 owing to its highest Laplace pressure. Furthermore, D4S0 showed lowest water harvesting performance as D4S0 has a low drainage efficiency as the distance between the conical structures is almost zero, which can hinder the water droplet drainage from the bottom surface. Thus, drainage efficiency should be considered as an important factor in water harvesting performance. For this reason, D3S1 exhibited the best performance (total collected average weight of 6.315 g in 10 min) although D4S0 had a larger surface area than D3S1 and D2S2 had a higher drainage efficiency than D3S1. These results indicate that when the number of conical structures is identical, a proper combination of the Laplace pressure, surface area, and drainage efficiency is important to achieve the best water harvesting performance. Therefore, when number of the conical structures was fixed, D3S1 showed the best performance for fog harvesting because of the proper combination of Laplace pressure and surface area, and drainage efficiency.

**Figure 6:**
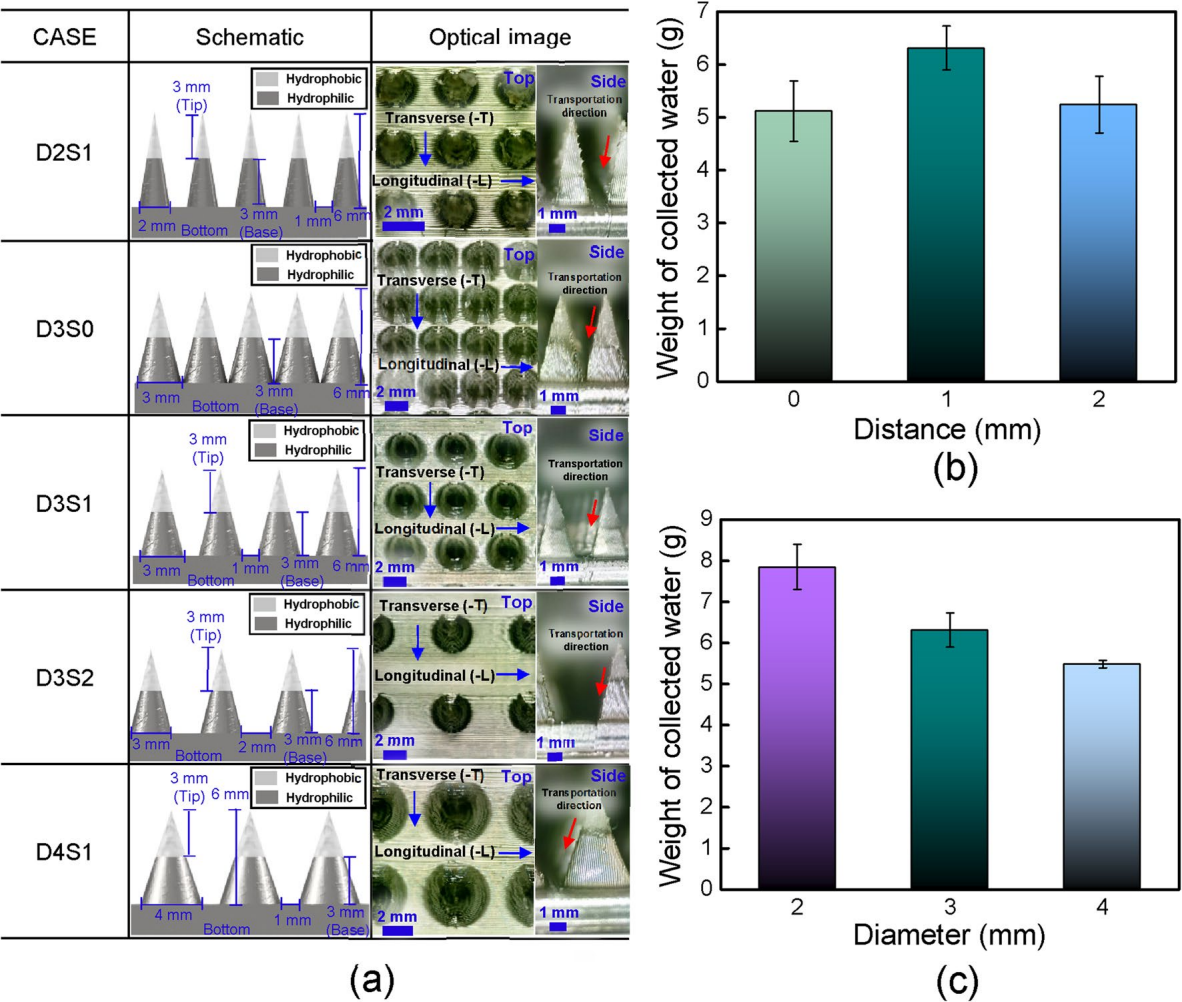
(**a**) Schematic and optical image (top view and side view) of BI-L with the different diameters of conical structure and distances between conical structures; (**b**) average amount of collected water for 10 min of using BI-L with fixed diameter of conical structure (3 mm); (**c**) average amount of collected water for 10 min of using BI-L with fixed distance between conical structures (1 mm).

To compare the performance of surfaces with fixed diameter of conical structures, three types of BI-L with different distances between conical structures and number of conical structures (i.e., D3S0, D3S1, and D3S2) were utilized, as shown in Fig. 6a. Despite significant differences in the number of conical structures, D3S1 showed the best performance (total collected average weight of 6.315 g in 10 min) compared to D3S0 and D3S2, as shown in Fig. 6b. At a fixed diameter of the conical structures, the surface area increased as the number of conical structures increased, whereas the distance between conical structures increased as the number of conical structures decreased. In addition, the water capture ability increased as the surface area increased, whereas the drainage efficiency decreased as the distance between conical structures decreased. Hence, although D3S0 has largest surface area, D3S0 exhibited the lowest water-harvesting performance owing to its low drainage efficiency, whereas D3S2 exhibited a slightly higher performance than D3S0 owing to its high drainage efficiency. Although D3S0 had a larger surface area than D3S1 and D3S2 had a higher drainage efficiency than D3S1, D3S1 exhibited the best performance as it has a proper combination between surface area and drainage efficiency. These results indicate that when the diameter of conical structures is fixed, although the importance of drainage efficiency and surface area in water harvesting is similar, a proper combination of the surface area and drainage efficiency is important to achieve the best water harvesting performance under the same Laplace pressure. Therefore, D3S1 also showed the best performance for fog harvesting because of the proper combination of surface area and drainage efficiency at a fixed diameter of the conical structures (3 mm).

To compare the performance of surfaces with fixed distance between conical structures and number of conical structures, three types of BI-L with different diameters and number of conical structures (i.e., D2S1, D3S1, and D4S1) were used, as depicted in Fig. 6a. When the distance between conical structures was fixed at 1 mm, D2S1 has the largest surface area because D2S1 has the largest number of conical structures compared to other surfaces. Furthermore, D2S1 has a highest Laplace pressure as the diameter of conical structures decreased. Therefore, D2S1 showed the best performance (total collected average weight of 7.85 g in 10 min) compared to other BI-L surfaces because of the proper combination of Laplace pressure and surface area, and drainage efficiency, as depicted in Fig. 6c.

## Conclusion

In this study, a hierarchically structured surface with microgrooved patterns was fabricated using FFF-type 3D printing. The proposed surface was designed to mimic the morphology of the cactus spine and dual wettability of the Namib Desert beetle. Microgrooved patterns of the cactus spine were implemented on the cactus-shaped PDMS surface using the staircase effect of FFF-type 3D printing. To realize the dual-wettability of Namib Desert beetles, a partial Pt deposition method using a paraffin wax-based masking method that enables mass production of complex morphology was introduced. Because of the surface energy gradient, although the hydrophobic region can condense more water droplets than the hydrophilic region, the condensed water in the hydrophobic region can be self-transported to the hydrophilic region. Furthermore, the cactus spine morphology enables condensed water droplets to self-transport from the tip to the base region using the Laplace pressure gradient. Moreover, the microgrooved pattern not only guides the water in a certain direction but also enhances surface wettability with an increase in surface area. To demonstrate the effects of conical structure and wettability in the fog-harvesting performance, qualitative and quantitative analyses were performed using six different types of surfaces under the same conditions. Consequently, the surfaces with conical structures exhibited higher fog harvesting performance than flat surfaces. It should be noted that the fog-harvesting performance of BI-L increased by approximately 123% compared to that of FO. Furthermore, among the surfaces with conical structure which has only difference with surface wettability condition, BI-L (D3S1) exhibited the highest performance (total collected average weight of 6.31 g in 10 min) using the fog-harvesting performance enhancement method (i.e., surface energy gradient, Laplace pressure gradient, hierarchical structure, microstructured layer-assisted water transportation, and gravitational force). In addition, the effects of three main factors (i.e., Laplace pressure, surface area, and drainage efficiency) in the fog-harvesting performance were demonstrated using BI-L with the variations of three main structural parameters (i.e., diameter of the conical structure, density of conical structures, and distance between conical structures). Note that, D2S1 exhibited the best performance (total collected average weight of 7.85 g in 10 min) compared with the other surfaces (D2S2, D3S0, D3S1, D3S2, D4S0, and D4S1) because of the proper combination of the Laplace pressure, surface area, and drainage efficiency. Namely, the fog harvesting performance of proposed surface can be more enhanced by newly deriving a proper combination of three main structural parameters (i.e., diameter of the conical structure, density of conical structures, and distance between conical structures). These results support an effective fog-harvesting surface that enables freshwater to be obtained even under harsh conditions, such as arid and polluted water environments.

## Supplementary Information

Supplementary Information.

## Data Availability

The datasets used and/or analyzed during the current study available from the corresponding author on reasonable request.
