# Supplementary material for: 3D-printing-assisted fabrication of hierarchically structured biomimetic surfaces with dual-wettability for water harvesting
Source: Sci Rep. 2023 Jul 1;13:10691. doi: 10.1038/s41598-023-37461-x (PMC10314913; doi:10.1038/s41598-023-37461-x)
Supplement: Supplementary file 1 — Supplementary Information. [file 41598_2023_37461_MOESM1_ESM.docx]

Supplementary Information

**3D-printing-assisted fabrication of hierarchically structured biomimetic surfaces with dual-wettability for water harvesting**

Yeongu Choi^1,†^, Keuntae Baek^1,†^, and Hongyun So^1,2,^*

^1^Department of Mechanical Engineering, Hanyang University, Seoul 04763, South Korea

^2^Institute of Nano Science and Technology, Hanyang University, Seoul 04763, South Korea

^†^These authors contributed equally

^*^Corresponding author. E-mail : hyso@hanyang.ac.kr

**I. Experimental setup**

To characterize and compare the water collection performances of the six different types of surfaces, an experimental setup that can prevent the influence of the external environment was established, as depicted in Fig. S1(a). An acrylic chamber was used to prevent the influence of the external environment and control the humidity inside the chamber. Notably, the internal environmental conditions of the chamber must be fixed when different types of surfaces are compared. A thermo-hygrometer (HTC-1, Tinyit) was used to monitor the humidity and temperature inside the chamber. Additionally, the temperature and relative humidity were almost fixed at 15 ℃ and 80%, respectively. The flow rate of moist air should be fixed for the same internal conditions when different types of surfaces are compared. Therefore, an ultrasonic humidifier (Miro-NR08, MIRO Co.) that can generate a constant moist airflow of 175 ml/h was used. The test bed was designed with a 45° slope so that water dripped only from the bottom on all surfaces. Furthermore, the center of the sample and the nozzle outlet were set to the same height. The distance between the center of the sample and the nozzle outlet was fixed at 60 mm, as illustrated in Fig. S1(b). It should be noted that the distance between humidifier and surfaces is set to 60 mm to minimize the time for fog harvesting experiment and to clearly compared fog harvesting performance with other surfaces, although the weight of collected water decreased as the distance between humidifier and surfaces increased, as shown in Fig. S2. The harvested water dripping from the bottom of the surface was collected in a Petri dish and measured using an electronic scale (A07, CHQ) with a resolution of 0.01 g.

**
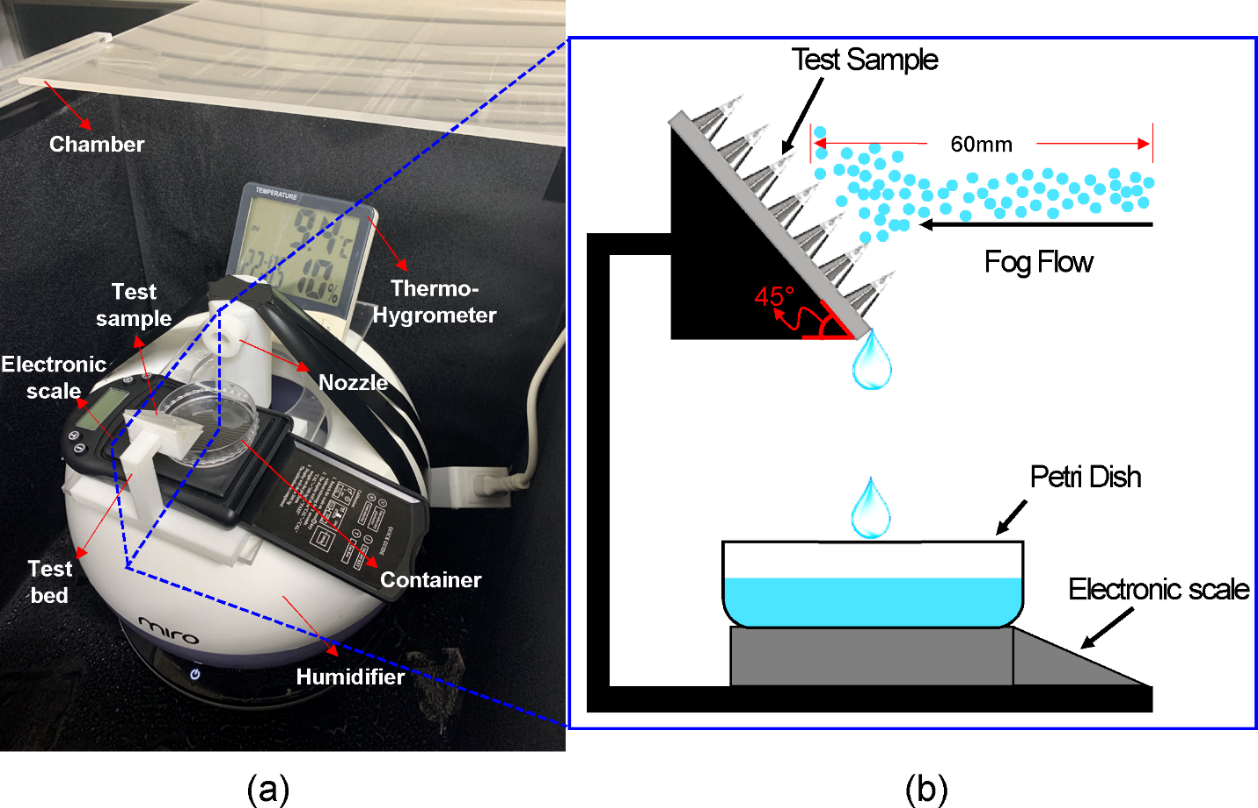
**

**Fig. S1** (a) Optical image of the experimental setup; (b) Schematic of the experimental setup (side view)


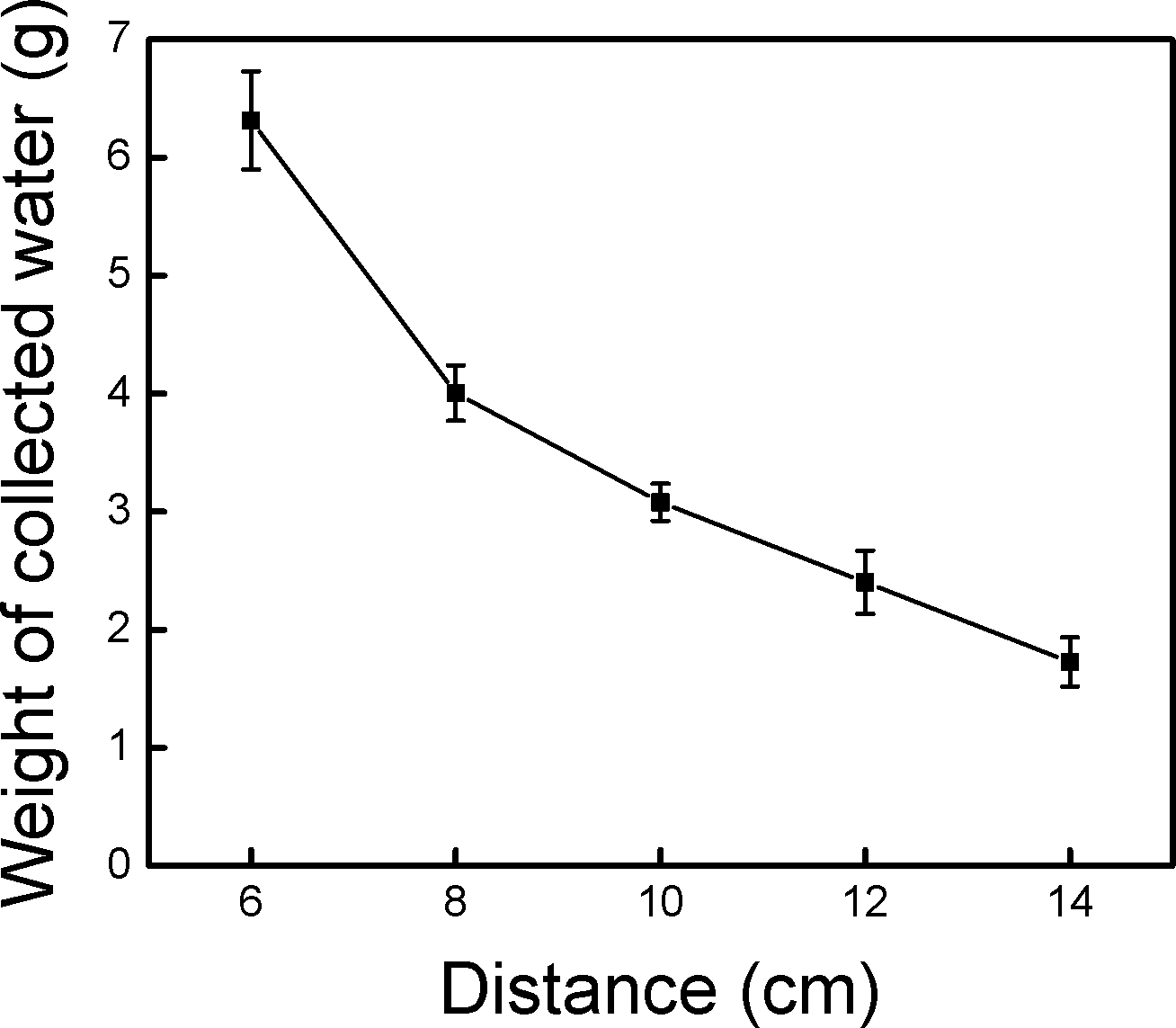


**Fig. S2** Comparison of water-collecting performance of BI-L for different distance between humidifier and surfaces.

**II. Surface analysis**

Qualitative and quantitative analyses were performed to demonstrate the water transportation and fog-harvesting performances of the proposed surfaces. A goniometer (Phoenix-MT(A), Surface Electro Optics, Korea) and an image analysis software (Image Pro 300) were used to measure the water contact angle (WCA) of the flat surfaces (i.e., FO and FI) and to characterize the moisture capturing morphologies of the four types of hierarchically structured surfaces (i.e., CO, CI, TI, and BI). Furthermore, optical images of the proposed surfaces were captured using a digital microscope (UM12; Vitiny) and high-resolution camera to analyze and characterize the moisture-capturing and transportation morphologies. The weight of the collected water was measured at least four times to obtain a reliable fog-harvesting performance of the proposed surface.

**III. Surface and structural characteristics**

Figure S3 shows the surface and structural properties using schematic and optical images of the six different types of surfaces (FO, FI, CO, CI, TI, and BI). FO and FI exhibited flat surfaces without any patterned structure (Figs. S3(a) and S3(b)). In contrast, the four different types of patterned surfaces (CO, CI, TI, and BI) have the same hierarchically structured pattern, as shown in Figs. S3(c), S3(d), S3(e), and S3(f). For the hierarchically structured surfaces, the detailed geometric parameters of the cone-shaped arrays, such as the height, diameter, and distance between two cones, were set to 6 mm, 3 mm, and 1 mm, respectively. It should be noted that even if only the macrostructure was designed, a macro-micro hierarchical structure was inevitably formed owing to the staircase effect of 3D printing technology. Therefore, the sharp microstructures that were transferred from the 3D printed mold with a printing angle of 80° can be observed in the optical images of the top and side views of the patterned surfaces (i.e., CO, CI, TI, and BI). Furthermore, because a staircase effect occurred during the layer-by-layer lamination process, the sharp microstructures were aligned in a certain direction. Thus, the longitudinal (L) and transverse (T) directions are parallel and orthogonal to the microstructured layer. The microstructured layer not only limits the transportation direction of the collected water but also increases the surface area. To modify the surface wettability, a hydrophobic surface was modified into a hydrophilic surface by introducing the Pt deposition method. In terms of flat surfaces, FO represents hydrophobicity with an average static WCA of 113.78° and FI represents hydrophilicity with an average static WCA of 38.91° owing to surface modification by Pt deposition, as listed in Table S2. In terms of patterned surfaces, CO was modified through Pt deposition to obtain three types of patterned surfaces (CI, TI, and BI). CI, TI, and BI can be classified according to the area where the surface modification was performed on CO. Among them, TI was prepared by partially depositing Pt on only the 3 mm tip part of the CO, while BI was prepared by depositing Pt in contrast to TI, as depicted in Figs. S3(e) and S3(f). Additionally, CI was obtained by depositing Pt on the entire CO surface. As previously mentioned, the microstructured layers on the patterned surfaces can increase the surface area. Thus, because the wettability characteristics can be enhanced by increasing the surface area, the difference between the hydrophobic and hydrophilic properties of a patterned surface with dual wettability (TI and BI) can be increased.

**
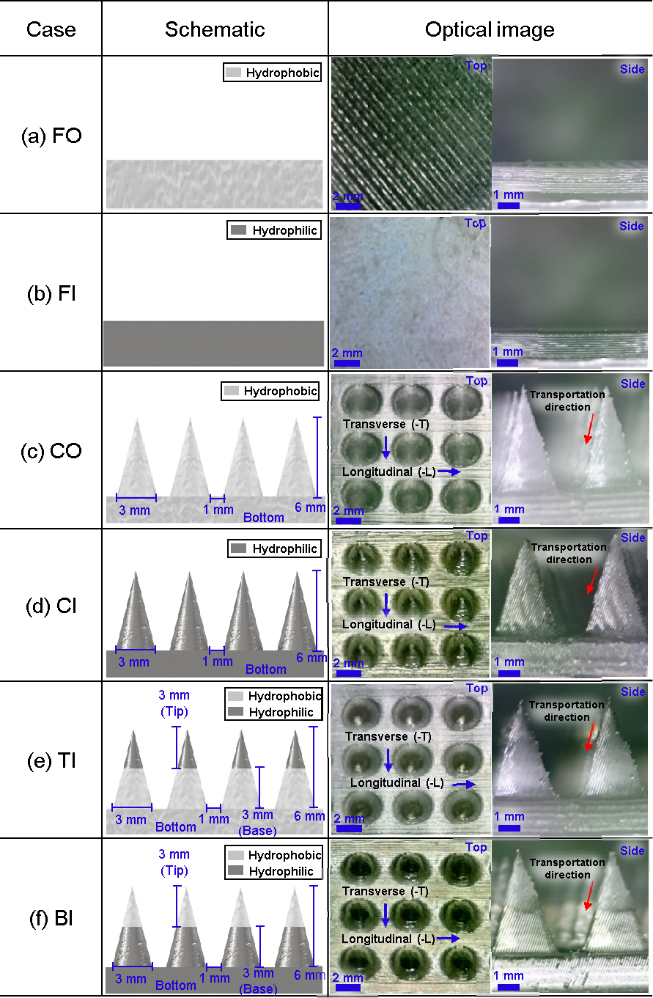
**

**Fig. S3** Schematic and optical image (top view and side view) of the six different surfaces

**
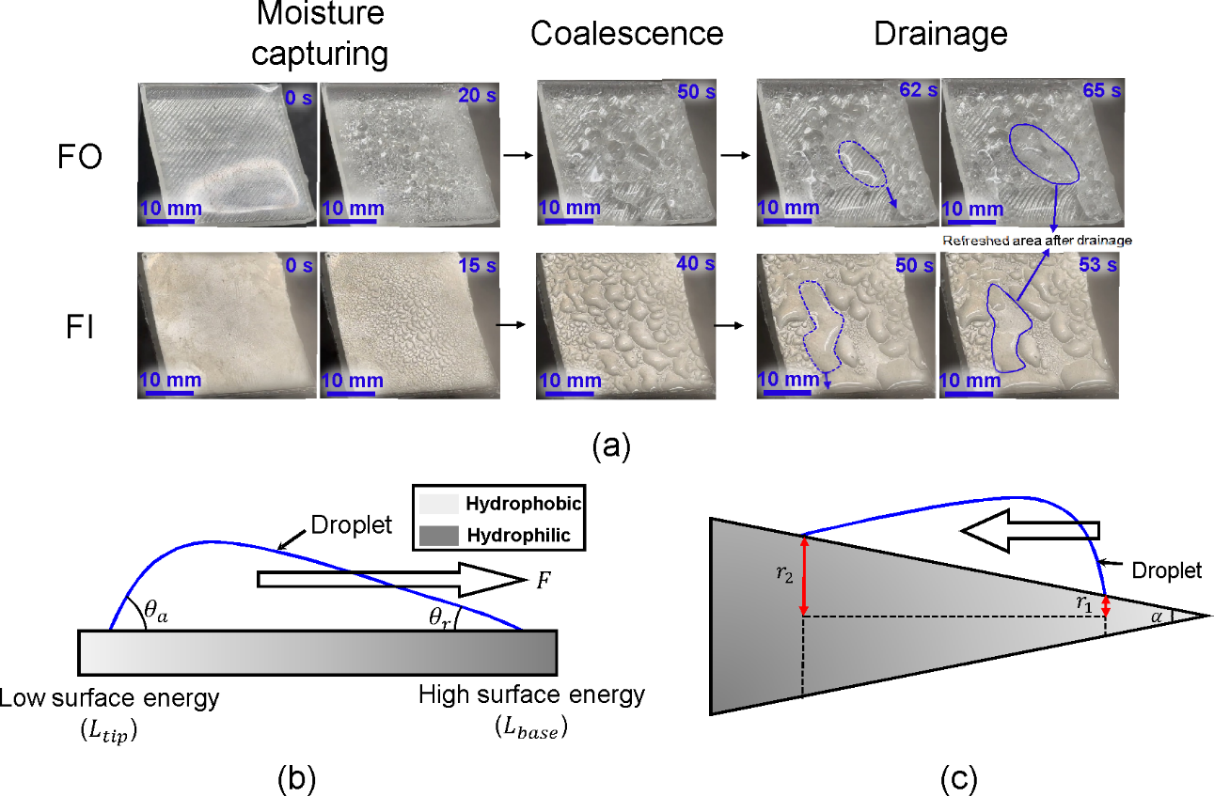
**

**Fig. S4** (a) Step-wise fog-harvesting behaviors (moisture capturing, coalescence, and drainage) on the flat surfaces; (b) Schematic of the surface energy gradient; (c) Schematic of the Laplace pressure gradient

**

**

**Fig. S5** Sequential zoomed images of the water-collecting process on the four different surfaces in two directions (-T and -L). (a) CO; (b) CI; (c) TI; (d) BI

**Table S1** Abbreviation of the six different surfaces for fog harvesting.

| **Abbreviation** | **Full name** |
| --- | --- |
| FO | **F**lat hydroph**o**bic PDMS surface without pattern |
| FI | **F**lat hydroph**i**lic PDMS surface without pattern |
| CO | **C**actus-shaped hydroph**o**bic PDMS surface with pattern |
| CI | **C**actus-shaped hydroph**i**lic PDMS surface with pattern |
| TI | Cactus-shaped and **t**ip hydroph**i**lic PDMS surface with pattern |
| BI | Cactus-shaped and **b**ase hydroph**i**lic PDMS surface with pattern |

**Table S2** Optical image and average WCA of the two different types of flat surfaces.

| **Type** | **WCA** | **Optical image** |
| --- | --- | --- |
| FO | 113.78° | 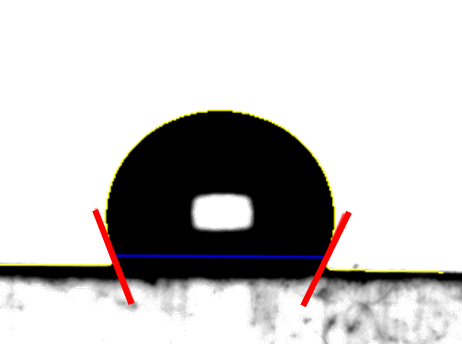 |
| FI | 38.91° | 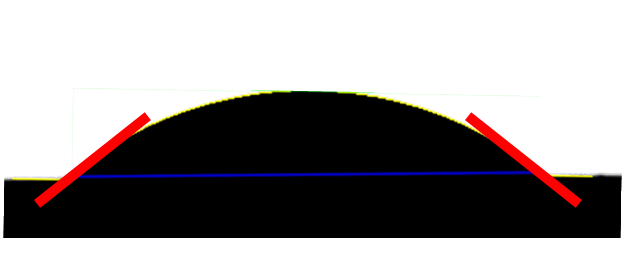 |

**Table S3** Geometry of seven different types of surfaces for fog harvesting.

| **Type** | **Diameter of conical structure** | **Distance between conical structures** | **Number of conical structures** |
| --- | --- | --- | --- |
| D2S1 | 2 mm | 1 mm | 81 |
| D2S2 | 2 mm | 2 mm | 49 |
| D3S0 | 3 mm | 0 mm | 81 |
| D3S1 | 3 mm | 1 mm | 49 |
| D3S2 | 3 mm | 2 mm | 25 |
| D4S0 | 4 mm | 0 mm | 49 |
| D4S1 | 4 mm | 1 mm | 25 |
